# Occurrence and Risk Factors of Infected Pancreatic Necrosis in Intensive Care Unit–Treated Patients with Necrotizing Severe Acute Pancreatitis

**DOI:** 10.1007/s11605-021-05033-x

**Published:** 2021-05-13

**Authors:** Henrik Leonard Husu, Miia Maaria Valkonen, Ari Kalevi Leppäniemi, Panu Juhani Mentula

**Affiliations:** 1 Department of Abdominal Surgery, University of Helsinki and Helsinki University Hospital, P.O. BOX 340, 00029 HUS Helsinki, Finland; 2 Intensive Care Medicine, Department of Perioperative, Intensive Care and Pain Medicine, University of Helsinki and Helsinki University Hospital, Helsinki, Finland

**Keywords:** Infected pancreatic necrosis, Intensive care unit, Multiple organ failure, Organ failure, Prophylactic antibiotics, Severe acute pancreatitis, Necrotizing pancreatitis

## Abstract

**Background:**

In patients with severe acute pancreatitis (SAP), infected pancreatic necrosis (IPN) is associated with a worsened outcome. We studied risk factors and consequences of IPN in patients with necrotizing SAP.

**Methods:**

The study consisted of a retrospective cohort of 163 consecutive patients treated for necrotizing SAP at a university hospital intensive care unit (ICU) between 2010 and 2018.

**Results:**

All patients had experienced at least one persistent organ failure and approximately 60% had multiple organ failure within the first 24 h from admission to the ICU. Forty-seven (28.8%) patients had IPN within 90 days. Independent risk factors for IPN were more extensive anatomical spread of necrotic collections (unilateral paracolic or retromesenteric (OR 5.7, 95% CI 1.5–21.1) and widespread (OR 21.8, 95% CI 6.1–77.8)) compared to local collections around the pancreas, postinterventional pancreatitis (OR 13.5, 95% CI 2.4–76.5), preceding bacteremia (OR 4.8, 95% CI 1.3–17.6), and preceding open abdomen treatment for abdominal compartment syndrome (OR 3.6, 95% CI 1.4–9.3). Patients with IPN had longer ICU and overall hospital lengths of stay, higher risk for necrosectomy, and higher readmission rate to ICU.

**Conclusions:**

Wide anatomical spread of necrotic collections, postinterventional etiology, preceding bacteremia, and preceding open abdomen treatment were identified as independent risk factors for IPN.

## Introduction

The incidence of infected pancreatic necrosis (IPN) correlates strongly with the prevalence of organ failures in acute pancreatitis.^1^ Development of IPN increases mortality risk and need for invasive interventions.^1^–^3^ Despite utilization of step-up treatment algorithm and implementation of minimally invasive debridement techniques, morbidity remains high in patients with IPN.^4^ Early enteral nutrition acts protective against IPN.^5^–^7^ Antibiotic prophylaxis has yet failed to show substantial clinical benefit in preventing IPN or diminishing associated mortality, and is thus recommended against treatment guidelines.^8^–^10^

Preoperative diagnosis of IPN can be difficult. Fever and increasing inflammation markers may indicate suspicion of IPN, but these are very common in patients with severe acute pancreatitis (SAP) treated in ICU; thus, additional risk factors for IPN may be useful in clinical decision-making. High rate of falsely negative fine needle aspirate and lack of clinical signs of infection can mislead clinician.^11^ Gas in necrotic collection on computed tomography is specific for infection but the sensitivity is low.^11^ Diagnostic interventions can cause bacterial contamination of otherwise sterile necrotic collections. Intervening in the natural course of a necrotic pancreatic collection is often based on clinical suspicion of infection. Ideally, diagnostic and therapeutic step-up interventions are targeted to patients with documented infection or with a high likelihood of IPN. If risk of IPN is low, drainage procedures should often be avoided. For these reasons, increasing knowledge on risk factors for IPN is important in clinical practice.

We studied the occurrence of IPN in a patient cohort with necrotizing SAP. The primary aim was to identify risk factors for IPN. The secondary aims were to report the morbidity and mortality of patients with IPN, and to identify late death risk factors for all patients in the study.

## Methods

### Patient Selection

This was a retrospective study of patients with necrotizing SAP. Patients with acute pancreatitis admitted within 7 days from hospital admission to Helsinki University Hospital ICU enrolled in the study. A systematic search of hospital records included all patients with the ICD-10 diagnosis code K85.X or K86.X treated at Meilahti Hospital ICU between January 1, 2010, and December 31, 2018. Edematous pancreatitis, acute-on-chronic pancreatitis, pancreatitis of a pancreas transplant, initial treatment abroad, or loss to follow-up excluded patients from the study.

### Data Collection

We gathered patient and survival data retrospectively from patient medical records. Survival data indirectly included (if needed) information provided by the Population Register Centre in Finland (http://www.vrk.fi). All patient data were merged into a separate database for this study purpose only. Replacement of patient identity information with running numbering protected the identity of patients. Conduction of study adhered to principles in STROBE statement (https://www.strobe-statement.org/).

### Definitions

Diagnosis of acute pancreatitis required fulfilling criteria of international guidelines.^8^ The Revised Atlanta Classification from 2012 graded acute pancreatitis severity.^12^ We collected renal, circulatory, and/or respiratory organ failures that occurred within 24 h from admission to ICU. A score of ≥ 2 according to the Modified Marshall Scoring System qualified as an organ failure.^12^ Categorization of hypoperfusion was according to the definitions of altered tissue perfusion and signs of shock by Cecconi et al.^13^ Study database included highest Sequential Organ Failure Assessment (SOFA) score within 72 h and highest Acute Physiology and Chronic Health Evaluation II (APACHE II) score within 24 h of intake to ICU.^14^,^15^ We calculated American Society of Anesthesiologists Physical Status Classification System (ASA) score to reflect pre-existing somatic disease burden of patients.^16^ Individual comorbidities were collected as mentioned in electronic health records. We recorded length of stay at the hospital and Helsinki University Hospital ICU. If patient was readmitted to ICU later than a week after discharge, it recorded as a readmission to the ICU.

Nonenhancement areas of the pancreas and/or the peripancreatic fat on contrast-enhanced CT scan characterized necrotizing pancreatitis. In the absence of such abdominal CT scan during the initial hospital stay (due to the risk of intravenous contrast medium–mediated kidney injury), we considered signs of necrotic pancreatitis on imaging studies at the outpatient clinic within 3 months following the insult of SAP, as evidence of endured necrotizing pancreatitis. When there was direct evidence of (peri)pancreatic necrosis during a surgical or endoscopic procedure, we classified the pancreatitis as necrotizing. We classified patients as having edematous pancreatitis if there were no (peri)pancreatic collections present on CT scan without contrast medium or on magnetic resonance imaging, or if patient did not undergo any imaging studies. Anatomical location of necrotic collections was collected as follows: central (around the pancreas) and peripheral (retromesenteric, left paracolic gutter, and right paracolic gutter). Patients with necrosis only around the pancreas served as reference when comparing to necrosis in one or multiple (widespread necrotic collection) of the peripheral locations.

A positive bacterial culture obtained preoperatively from a pancreatic or peripancreatic tissue aspirate or drainage, or a positive bacterial tissue culture from the first necrosectomy operation, characterized as infected pancreatic necrosis. The presence of a positive blood culture sample defined bacteremia. Blood-cultured common skin contaminants required at least two different positive samples for the diagnosis of bacteremia. The Guidelines by the American Thoracic Society and Infectious Diseases Society of America directed collection of data on hospital-acquired pneumonias (including ventilator-acquired pneumonia).^17^ An infectious disease specialist reviewed all cases of suspected pneumonia.

### Institutional Treatment Protocol

The ICU is a mixed surgical-medical ICU treating approximately 1800 patients per year. Patients received a 5-day antibiotic prophylaxis (cefuroxime 1.5g three times a day, starting on day 0 at the ICU). Antimicrobial prophylaxis protocol remained unchanged during the study period. Treatment strategy was to avoid any interventions towards necrotic collections whenever possible. When intervention was clinically deemed inevitable, percutaneous and/or endoscopic drainage was, when feasible, the first-line treatment, followed by open necrosectomy only when upstaging procedures were clinically unavoidable. Patients with ongoing open abdomen for abdominal compartment syndrome were not considered candidates for step-up treatment strategy, rather open necrosectomy was pursued in these patients if postponing intervention was not feasible.

### Statistical Analysis

We used Excel (Microsoft Corp., Redmond, WA, USA) and SPSS 25.0 (IBM Corp., Armonk, USA) to analyze the acquired data. Significance level was set at 5%. Statistical testing of proportions utilized Fisher’s exact two-sided test. Comparison of length of stay was implemented with the Mann-Whitney *U* test.

For the primary aim, we performed a univariate analysis of a pre-specified list of variables for IPN by using binary logistic regression models. In multivariable binary logistic regression model, we included all variables with a *p* value equal to or less than 0.05 in the univariate analysis. From the univariate and the multivariable binary logistic regression models, we calculated odds ratios (OR) with 95 percent confidence intervals (CI) for developing IPN.

For the secondary aims, we report morbidity and mortality of patients with and without IPN. We used the Kaplan-Meier method for the estimation of survival for patients with and without IPN and presented the results graphically with survival curves. For all study patients, we performed a univariate analysis of a pre-specified list of variables for death between days 8 and 90 after admission to ICU using binary logistic regression models.

## Results

**Fig. 1:**
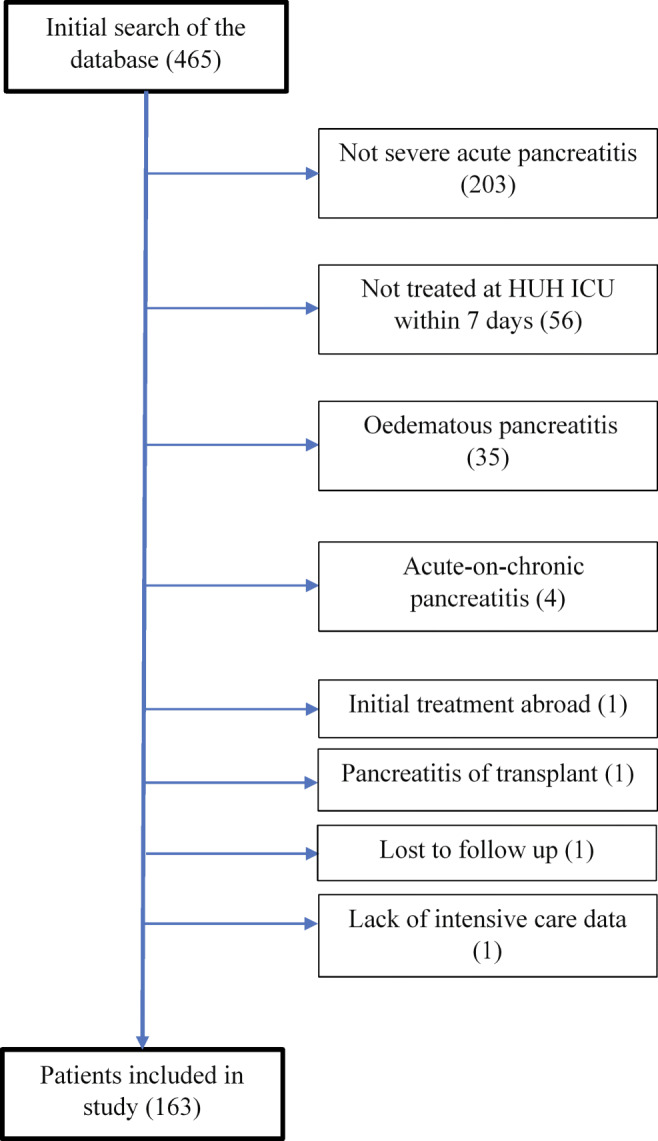
Flow chart

The flow chart (Fig. 1) visualizes the acquisition of patients (*n* = 163). Around two-thirds of all patients (111 patients or 68.1%) had alcoholic necrotizing pancreatitis. Table 1 summarizes all infections within 90 days. Pneumonia, bacteremia, and IPN occurred at a respective median of 4 (IQR 1–11), 16 (IQR 1–52), and 23 (IQR 19–33) days after ICU admission.

**Table 1:** Infections within 90 days from intensive care unit admission, patients with necrotizing pancreatitis treated between years 2010 and 2018 (*n* = 163)

	*n* (%)
Any infectious complication	84 (51.5)
Bacteremia, pneumonia, or infected pancreatic necrosis	72 (44.2)
Bacteremia*	21 (12.9)
Pneumonia	21 (12.9)
Infected pancreatic necrosis	47 (28.8)
Other	31 (19.0)
Intra-abdominal	12 (7.4)
Postoperative	7 (4.3)
Perforation of intestine	4 (2.5)
Ischemia, translocation	1 (0.6)
*Clostridium difficile* enterocolitis	7 (4.3)
Biliary†	4 (2.5)
Infected pseudocyst	3 (1.8)
Urinary tract infection (not septic)	4 (2.5)
Catheter-related infection (not septic)	3 (1.8)
Surgical site infection (wound)	3 (1.8)
Aortic prosthesis infection	1 (0.6)
Endocarditis	1 (0.6)
Perianal abscess	1 (0.6)

This table summarizes the total number of diagnosed infections. Some patients suffered from more than one infection

*Following infected pancreatic necrosis (6), following urinary tract infection (3), and catheter-related (2)

†Acalculous/calculous cholecystitis and/or cholangitis

**Fig. 2:**
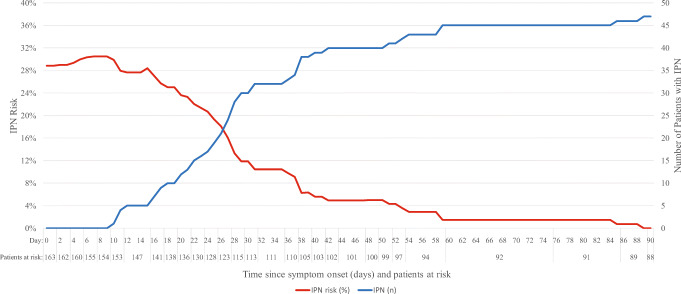
Cumulative number of patients with infected pancreatic necrosis (IPN) and risk of IPN within 90 days

Forty-seven (28.8%) patients had IPN within 90 days since the first admission to ICU. The highest levels in the risk of IPN were seen during the first week since admission to ICU (Fig. 2). Due to a steep decrease after the first week, the risk reduced approximately by half by the end of the first month. Table 2 presents the results of the univariate analysis of the potential risk factors for IPN. Postinterventional (postoperative or post-ERCP) etiology included 5 patients with pancreatitis following a surgical procedure (none of whom underwent pancreatic surgery), 4 patients with post-ERCP pancreatitis, and 1 patient with pancreatitis following endoscopic papillectomy. Based on the results of the multivariable logistic regression model (Table 3), several factors were associated with higher odds of developing IPN. As compared to patients with alcoholic necrotizing pancreatitis, patients with postoperative or post-ERCP pancreatitis were more likely to develop IPN (OR 13.5, 95% CI 2.4–76.5). We observed higher odds of developing IPN for patients with widespread necrotic collections (unilateral paracolic or retromesenteric [OR 5.7, 95% CI 1.5–21.1] and bilateral paracolic or unilateral paracolic and retromesenteric [OR 21.8, 95% CI 6.1–77.8]) relative to those with central location of the necrotic collections, as well as for patients with preceding bacteremia (OR 4.8, 95% CI 1.3–17.6) relative to patients without bacteremia, and for patients with open abdomen treatment for abdominal compartment syndrome (OR 3.6, 95% CI 1.1–9.3) relative to those without this treatment.

**Table 2:** Baseline characteristics of patients with and without IPN and crude odds ratios with 95% confidence intervals for developing IPN calculated from the univariate logistic regression models (*N* = 163)

	No IPN (*n* = 116)	IPN (*n* = 47)	OR (95% CI)	*p*
Age, median (range), years	50 (18–82)	51 (25–66)	1.00 (0.97–1.02)	0.682
BMI, mean ± SD, kg/m^2^	29.6 ± 5.1	29.1 ± 4.1	0.98 (0.91–1.05)	0.560
Male sex	93 (80.2)	40 (85.1)	1.41 (0.56–3.56)	0.512
ASA classification ≥ III at hospital admission	61 (52.6)	25 (53.2)	1.03 (0.52–2.02)	1.000
Tertiary referral	36 (31.0)	20 (42.6)	1.65 (0.82–3.31)	0.203
Any somatic comorbidity*	37 (31.9)	11 (23.4)	0.65 (0.30–1.42)	0.345
Etiology				
Alcoholic	80 (69.0)	31 (66.0)	Reference	
Postoperative and post-ERCP†	2 (1.7)	8 (17.0)	10.32 (2.08–51.33)	0.004
Biliary	23 (19.8)	5 (10.6)	0.56 (0.20–1.61)	0.282
Other	11 (9.5)	3 (6.4)	0.70 (0.18–2.69)	0.608
Decreased tissue perfusion < 24h from ICU admission	89 (76.7)	41 (87.2)	2.07 (0.80–5.41)	0.196
Organ failure < 24h from ICU admission‡				
Respiratory failure	112 (96.6)	47 (100.0)	NC	0.325
Circulatory failure	61 (52.6)	31 (66.0)	1.75 (0.86–3.54)	0.163
Renal failure	37 (31.9)	19 (40.4)	1.45 (0.72–2.92)	0.363
Multiple organ failure	70 (60.3)	32 (68.1)	1.40 (0.68–2.87)	0.378
APACHE II < 24 h from ICU admission||	16 (13–23)	19 (12–23)	1.00 (0.96–1.04)	0.968
Highest SOFA < 72 h from ICU admission||	9 (6–11)	10 (7–13)	1.08 (0.99–1.17)	0.086
Bacteremia§	8 (6.9)	7 (14.9)	2.36 (0.80–6.94)	0.136
Pneumonia§	17 (14.7)	3 (6.4)	0.40 (0.11–1.43)	0.191
Open abdomen§	21 (18.1)	18 (38.3)	2.81 (1.32–5.97)	0.008
Pancreatic parenchymal necrosis > 50% on CT ¶	9 (13.2)	5 (20.0)	1.64 (0.49–5.47)	0.514
Anatomical location of the necrotic collection				
Central (around the pancreas)	56 (48.3)	5 (10.6)	Reference	
Unilateral paracolic or retromesenteric	35 (30.2)	12 (25.5)	3.84 (1.25–11.83)	0.019
Widespread**	25 (21.6)	30 (63.8)	13.44 (4.67–38.70)	< 0.001

Presented values are absolute number of patients (numbers in parenthesis represent percentages) unless stated otherwise

*Heart disease (e.g., coronary artery disease), pulmonary disease (e.g., chronic obstructive pulmonary disease), chronic renal insufficiency, liver cirrhosis, and/or diabetes

†One patient with severe acute pancreatitis following endoscopic papillectomy was included in this group

‡According to Modified Marshall Scoring System. Reference category is patients without the organ failure in question

||Median (interquartile range)

§Events following diagnosis of infected pancreatic necrosis were discarded

¶Proportion of pancreatic necrosis on CT not evaluable in 70 patients (22 with IPN), thus *n* = 93

**Bilateral paracolic or unilateral paracolic and retromesenteric

*APACHE II*, Acute Physiology and Chronic Health Evaluation II Scoring System; *ASA*, American Society of Anesthesiologists Physical Status Classification System; *BMI*, body mass index; *CI*, confidence interval; *CT*, computed tomography; *ERCP*, endoscopic retrograde cholangiopancreatography; *ICU*, intensive care unit; *IPN*, infected pancreatic necrosis; *NC*, not countable; *OR*, odds ratio; *SD*, standard deviation; *SOFA*, Sequential Organ Failure Assessment score

**Table 3:** Odds ratios and 95% confidence intervals for developing infected pancreatic necrosis calculated from the multivariable logistic regression model (*N* = 163)

	OR (95% CI)	*p*
Anatomical location of the necrotic collection		
Central (around the pancreas)	Reference	
Unilateral paracolic or retromesenteric	5.67 (1.52–21.09)	0.010
Widespread*	21.75 (6.08–77.81)	< 0.001
Etiology		
Alcoholic	Reference	
Postoperative and post-ERCP†	13.47 (2.38–76.45)	0.003
Biliary	1.21 (0.34–4.29)	0.765
Other	0.52 (0.11–2.49)	0.409
Bacteremia‡	4.82 (1.31–17.64)	0.018
Open abdomen‡	3.62 (1.41–9.29)	0.007

Backward conditional logistic regression model of variables with *p* ≤ 0.20 in Table 2. The variable “Decreased tissue perfusion < 24h from ICU admission” was not entered in the equation due to risk of collinearity. The variable “Highest SOFA < 72h from ICU admission” was not entered in the equation as no clinically meaningful cutoff value could be demonstrated (AUROC 0.597)

*Bilateral paracolic or unilateral paracolic and retromesenteric

†One patient with severe acute pancreatitis following endoscopic papillectomy was included in this group

‡Events following diagnosis of IPN were discarded

*AUROC*, area under the receiver operating characteristics; *CI*, confidence interval; *ERCP*, endoscopic retrograde cholangiopancreatography; *ICU*, intensive care unit; *OR*, odds ratio; *SOFA*, Sequential Organ Failure Assessment score

**Fig. 3:**
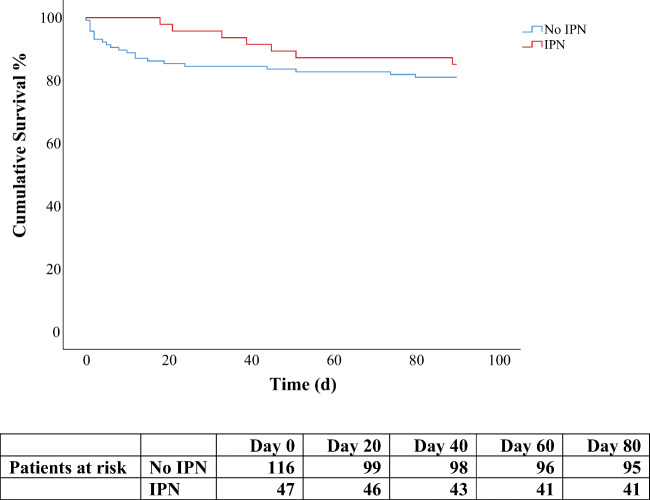
Kaplan-Meier survival estimate for patients with and without IPN

By 90 days, 29 (17.8%) patients died. Of those with and without IPN, 7 (14.9%) and 22 (19.0%) patients died, respectively. Survival curves of patients with and without IPN demonstrated patterns diverging already from the start of ICU treatment period (Fig. 3). Risk factors for death after the first week of treatment were related to previous health status, disease severity, and open abdomen treatment (Table 4).

**Table 4:** Univariate logistic regression analysis of potential risk factors for late death (*N* = 152)

	Survivors (*n* = 134)	Non-survivors (*n* = 18)	OR (95% CI)	*p*
Age, median (range), years	50 (18–77)	60 (38–65)	1.04 (1.00–1.08)	0.090
BMI, mean ± SD, kg/m^2^	29.4 ± 4.6	29.1 ± 5.6	0.99 (0.89–1.10	0.790
Male sex	110 (82.1)	16 (88.9)	1.75 (0.38–8.10)	0.740
ASA classification ≥ III at hospital admission	63 (47.0)	16 (88.9)	9.02 (1.99–40.76)	< 0.001
Tertiary referral	46 (34.3)	7 (38.9)	1.22 (0.44–3.35)	0.794
Any somatic comorbidity*	34 (25.4)	11 (61.1)	4.62 (1.66–12.87)	0.004
Etiology				
Alcoholic	93 (69.4)	13 (72.2)	Reference	
Postoperative and post-ERCP†	8 (6.0)	1 (5.6)	0.89 (0.10–7.74)	0.919
Biliary	23 (17.2)	0 (0.0)	0.00 (0.00–NC)	0.998
Other	10 (7.5)	4 (22.2)	2.86 (0.78–10.47)	0.112
Decreased tissue perfusion < 24h from ICU admission	101 (75.4)	18 (100.0)	NC	0.013
Organ failure < 24h from ICU admission‡				
Respiratory failure	130 (97.0)	18 (100.0)	NC	1.000
Circulatory failure	65 (48.5)	17 (94.4)	18.05 (2.34–139.49)	< 0.001
Renal failure	35 (26.1)	11 (61.1)	4.45 (1.60–12.36)	0.005
Multiple organ failure	74 (55.2)	17 (94.4)	13.78 (1.78–106.57)	0.001
APACHE II < 24h from ICU admission||	15 (12–20)	23 (21–28)	1.18 (1.09–1.29)	< 0.001
Highest SOFA < 72h from ICU admission||	8 (5–11)	13 (8–17)	1.35 (1.16–1.57)	< 0.001
Bacteremia	15 (11.2)	5 (27.8)	3.05 (0.95–9.76)	0.065
Pneumonia	19 (14.2)	0 (0.0)	NC	0.130
IPN	40 (29.9)	7 (38.9)	1.50 (0.54–4.14)	0.428
Open abdomen	26 (19.4)	12 (66.7)	8.31 (2.85–24.21)	<.001
Pancreatic parenchymal necrosis > 50% on CT§	13 (14.6)	1 (33.3)	2.92 (0.25–34.61)	0.394
Anatomical location of the necrotic collection				
Central (around the pancreas)	48 (35.8)	5 (27.8)	Reference	
Unilateral paracolic or retromesenteric	43 (32.1)	3 (16.7)	0.67 (0.15–2.97)	0.598
Widespread¶	43 (32.1)	10 (55.6)	2.23 (0.71–7.05)	0.171

Analysis of potential risk factors for death that occurred between days 8 and 90 days after ICU admission. Presented values are absolute number of patients (numbers in parenthesis represent percentages) unless stated otherwise

*Heart disease (e.g., coronary artery disease), pulmonary disease (e.g., chronic obstructive pulmonary disease), chronic renal insufficiency, liver cirrhosis, and/or diabetes

†One patient with severe acute pancreatitis following endoscopic papillectomy was included in this group

‡According to Modified Marshall Scoring System

||Median (interquartile range)

§Proportion of pancreatic necrosis on CT not evaluable in 60 patients (15 that died), thus *n* = 92

¶Bilateral paracolic or unilateral paracolic and retromesenteric

*APACHE II*, Acute Physiology and Chronic Health Evaluation II Scoring System; *ASA*, American Society of Anesthesiologists Physical Status Classification System; *BMI*, body mass index; *CI*, confidence interval; *CT*, computed tomography; *ERCP*, endoscopic retrograde cholangiopancreatography; *ICU*, intensive care unit; *IPN*, infected pancreatic necrosis; *NC*, not countable; *OR*, odds ratio; *SOFA*, Sequential Organ Failure Assessment score

In the analysis of factors related to patient morbidity, significant differences were observed depending on IPN status. Patients with IPN had a longer length of stay in the ICU (median 31 vs 8 days, *p* < 0.001) and a longer overall length of hospital stay (median 69 vs 21 days, *p* < 0.001). In patients surviving the initial ICU treatment period (*n* = 143 of whom 42 had IPN), readmission to ICU was significantly more frequent in patients with IPN (14 patients or 33.3%) compared to patients without IPN (1 patient or 1.0%, *p* < 0.001). Open necrosectomy was utilized more frequently in patients with IPN (43 of 47 patients, 91.5%) than in patients without IPN (6 of 116 patients, 5.2%; *p* < 0.001). Treatment of necrotic collections is presented in Table 5.

**Table 5:** Interventions for necrotic collections (*N* = 163)

	No IPN (*n* = 116)	IPN (*n* = 47)	*p*
No intervention	99 (85.3)	0 (0.0)	< 0.001
Drainage without debridement (endoscopic/percutaneous)	11 (9.5)	4 (8.5)	1.000
Open necrosectomy	6 (5.2)	43 (91.5)	< 0.001
Preceding drainage (endoscopic/percutaneous)*	1 (16.7)	20 (46.5)	
Ongoing open abdomen at the time of necrosectomy*	1 (16.7)	14 (32.6)	
Emergent other indication for laparotomy*	2 (33.3)	3 (7.0)	
Lack of safe percutaneous drainage route*	1 (16.7)	1 (2.3)	
Step-up treatment not considered*†	1 (16.7)	5 (11.6)	

Interventions for necrotic collections within 90 days from admission to ICU. Presented values are absolute number of patients (numbers in parenthesis represent percentages). *p* value is Fisher’s exact 2-sided test result

*Percentage of patients that underwent necrosectomy (in parenthesis)

†Treating surgeons’ choice to proceed with open necrosectomy without preceding drainage

*CI*, confidence interval; *IPN*, infected pancreatic necrosis; *NC*, not countable; *OR*, odds ratio

## Discussion

This study reports risk factors and outcome for IPN in ICU-treated patients with necrotizing SAP. Infected pancreatic necrosis presented in more than a quarter of patients. We report here evidence of an increased risk of IPN in patients with wide anatomical spread of necrotic collections, postinterventional etiology of pancreatitis, preceding bacteremia, and open abdomen treatment. IPN was associated with significant morbidity compared to patients without IPN.

The proportion of patients with IPN that we report here is within the range of previous studies.^1^ The strength of the present study was to include only patients with persistent organ failure and admission to ICU in the early disease course. We observed significant morbidity related to IPN in terms of length of stay, need for ICU resources, and need for necrosectomy. We also observed that around half of ICU-treated patients with necrotizing SAP die without documented IPN. Recent results from a large prospective multicenter study support our study findings, as IPN was associated with significant morbidity, but not mortality, compared to patients with sterile necrotic collections, when adjusting for persistent organ failure.^18^ Preceding health status and disease severeness were associated with late death risk of patients in our study.

In the present study, around 60% of IPN occurred within the first 4 weeks after symptom debut of SAP, and almost half of all cases of IPN were diagnosed during the third or fourth week of the disease. In line with these findings, previous studies have shown that a significant portion of patients develop IPN within the first weeks of disease.^19^ Although postponing intervention up to 4 weeks is generally advocated in guidelines,^8^,^9^ it should be individually evaluated if persisting physiological disturbances or deterioration is presumed to be maintained by early IPN. Especially as encapsulation of necrotic collections seems to occur earlier in a significant percentage of patients.^20^

Institutional protocol mandated short antibiotic prophylaxis within the study period. With this treatment policy, we observed that IPN occurred in less than 30% of patients. A markedly higher percentage of patients with IPN has been reported from studies using total parenteral nutrition^21^,^22^ and in some studies without prophylactic antimicrobials.^2^,^23^ In contrast, lower or corresponding frequencies of IPN have been found in some studies using on-demand or similar antibiotic protocol to ours, but these studies also included patients with mainly a less severe degree of acute pancreatitis.^24^–^26^ The present study was not designed to evaluate the effects of prophylactic antibiotic treatment in necrotizing SAP. However, the observed high number of infections despite the use of prophylactic antibiotics raises questions about the utility of a prophylactic antibiotic protocol. The authors recommend following the existing treatment guidelines recommending against the use of antibiotic prophylaxis.^8^–^10^

This study reports an independent association between postoperative or post-endoscopic etiology and the risk of IPN. Although post-ERCP pancreatitis rarely becomes severe,^27^ IPN is common in severe post-ERCP pancreatitis according to results from the present study. Necrotizing SAP in this subgroup of patients might be fuelled by bacterial infection; however, magnitude of the odds ratio should be interpreted very cautiously due to the low number of events.

As our results show, wide anatomical spread of necrotic collections increases the risk of IPN. We found three previous studies reporting an association between measured extra-pancreatic necrosis volume and IPN in acute pancreatitis^28^–^30^ that included patients with mild edematous pancreatitis,^28^–^30^ interpreted certain edematous radiological findings as necrotic,^29^ or defined infection unconventionally.^28^ A recent analysis of patients with only necrotic SAP found the number of necrotic collections independently associating with the risk of IPN.^31^ Results from these previous reports and those of the present study warrant clinician to consider widespread necrotic collections as if they are infected with low threshold. An increased proportion of pancreatic parenchymal necrosis has previously been associated with an increased risk of IPN, but our study failed to show such association.^2^,^32^

Open abdomen treatment was associated with an increased risk of IPN. The association between preceding open abdomen treatment and increased risk of IPN in SAP has not been previously reported. Open abdomen treatment due to refractory abdominal compartment syndrome is a last but unavoidable measure preserved for patients that suffer from the severest form of acute pancreatitis. Presumably the severe disease itself is a leading cause of the increased susceptibility of IPN. Shattering the sterile intraperitoneal space might increase the risk of direct or indirect bacterial translocation to the retroperitoneal space.

As is shown by our study, IPN results in an increasingly morbid outcome for the patient. In attempting to decrease the rate of IPN, efforts to identify and treat incipient organ failure with subsequent low threshold for admission to ICU becomes essential. Adequate and early treatment of hypovolemia and developing shock is best accomplished in ICU settings with enough monitoring and treatment capacity of developing organ failures. Optimization of patient outcome should include early preventive conservative management of intra-abdominal hypertension, as abdominal compartment syndrome with consequent open abdomen treatment was significantly associated with IPN in the present study. Empirical early antibiotic treatment is justifiable in the most severely ill patients (e.g., patients with high SOFA score) if clinical suspicion of possible underlying concurrent infectious etiology of shock arises.

This study has weaknesses. The retrospective setting with a prospectively uncontrolled patient cohort, non-standardized treatment protocol, and possible differences in the specifics regarding antibiotic treatment might introduce some bias to this study. In the present study, a reliable evaluation of potential difference in mortality between patients with and without IPN cannot be performed because of the study design. In fact, patients with IPN had to survive until IPN was developed, whereas patients without IPN included those who died early (and did not even had chance to develop IPN). This is known as survival bias. Therefore, those with and without IPN differed by their risk of death already before IPN was developed. This study cannot provide a reliable estimate of the difference in mortality attributable to IPN itself. Another weakness is that for some of the studied potential risk factors there were quite small number of patients who presented with these conditions of interest. Thus, it is possible that some of the observed associations are due to chance only or some of the observed differences are under- or overestimated.

## Conclusions

More than a quarter of patients with necrotizing SAP develop IPN despite short antibiotic prophylaxis. Widespread necrotic collections, postinterventional etiology, preceding bacteremia, and open abdomen treatment were independently associated with an increased risk for IPN. A significantly morbid outcome was associated with IPN.

## Declarations

The Department of Abdominal Surgery at Helsinki University Hospital approved the conduction of the study.

## Abbreviations

APACHE II: Acute Physiology and Chronic Health Evaluation II (score)
ASA: American Society of Anesthesiologists Physical Status Classification System
BMI: body mass index
CI: confidence interval
CRP: C-reactive protein
CT: computed tomography
ERCP: endoscopic retrograde cholangiopancreatography
ICU: intensive care unit
IPN: infected pancreatic necrosis
IQR: interquartile range
KDIGO: Kidney Disease Improving Global Outcomes
MRI: magnetic resonance imaging
OR: odds ratio
SAP: severe acute pancreatitis
SD: standard deviation
SOFA: Sequential Organ Failure Assessment (score)
WBC: white blood cell
